# What Severe Medication Errors Reported to Health Care Supervisory Authority Tell About Medication Safety?

**DOI:** 10.1097/PTS.0000000000000914

**Published:** 2021-09-27

**Authors:** Carita Linden-Lahti, Anna Takala, Anna-Riia Holmström, Marja Airaksinen

**Affiliations:** From the ∗ Helsinki University Hospital (HUS), HUS Pharmacy; † Division of Pharmacology and Pharmacotherapy, Faculty of Pharmacy, University of Helsinki, Helsinki, Finland.

**Keywords:** patient safety, medication safety, severe medication error, health care supervisory authority

## Abstract

**Objectives:**

This study investigated severe medication errors (MEs) reported to the National Supervisory Authority for Welfare and Health (Valvira) in Finland and evaluated how the incident documentation applies to learning from errors.

**Methods:**

This study was a retrospective document analysis consisting of medication-related complaints and authoritative statements investigated by Valvira in 2013 to 2017 (n = 58).

**Results:**

Medication errors caused death or severe harm in 52% (n = 30) of the cases (n = 58). The majority (83%; n = 48) of the incidents concerned patients older than 60 years. Most likely, the errors occurred in prescribing (n = 38; 47%), followed by administration (n = 15; 19%) and monitoring (n = 14; 17%). The error process often included many failures (n = 24; 41%) or more than one health professional (n = 16; 28%). Antithrombotic agents (n = 17; 13%), opioids (n = 10; 8%), and antipsychotics (n = 10; 8%) were the therapeutic groups most commonly involved in the errors. Almost all error cases (91%; n = 53) were assessed as likely or potentially preventable. In 60% (n = 35) of the cases, the organization reported actions taken to improve medication safety after the occurrence of the investigated incident.

**Conclusions:**

Medication errors reported to the national health care supervisory authority provide a valuable source of risk information and should be used for learning from severe errors at the level of health care systems. High age remains a key risk factor to severe MEs, which may be associated with a wide range of medications including those not typically perceived as high-alert medications or high-risk administration routes. Despite being complex processes, the severe MEs have a great potential to lead to developing systems, processes, resources, and competencies of health care organizations.

Medication errors (MEs) are one of the most prevalent types of errors in patient care.^1,2^ The reduction of severe MEs should be a primary target of medication risk management in health care systems.^3^ This concept is also promoted by current global actions, such as the World Health Organization third patient safety challenge aiming to reduce ME-associated deaths by 50% by the year 2022.^4^ The definitions of severe patient safety incidents vary greatly,^5^ and in this article, severe MEs refer to the errors that are causing severe harm or have the potential to cause severe harm.

Previous studies on severe MEs have demonstrated challenges in several phases of the medication process, especially in prescribing and follow-up, administration, and patient transitions.^4,6–13^ The patient groups most vulnerable to severe MEs and harm have been pediatric patients and patients with comorbidities, polypharmacy, and high age.^6,7,13–15^ Also, high-alert medications causing more likely severe harm to patients, if associated with the error, have been identified and recommended to be prioritized in the development of medication safety.^3,6,7,13,15–19^

Medication error reporting systems (MERs) are among the recommended actions to learn from errors and near misses in health care.^1,20–23^ However, comprehensive national or even local MER systems are not established in many countries.^22,24^ There are also other challenges associated with MER systems, such as underreporting and the quality of retrospective data.^21,23^ Furthermore, these systems rarely capture severe, fatal errors.^25^ Therefore, MER systems should be complemented with other data sources and methods for medication risk management.^21^ Health care authority or patient insurance-based safety incident databases could serve as valuable data sources in this respect.^26^ However, these databases have been underused, although some evidence exists on their successful use in medication risk management research.^9,26–30^

Finnish health care system is based on public services available for all citizens. Municipalities are responsible for organizing and financing the services. The care is arranged in primary care (e.g., hospitals and public care centers), secondary and tertiary care (central and university hospitals), and social care units (e.g., assisted living facilities and home care for elderly or disabled people). Private sector produces only a quarter of the health care services. In Finland, systematic patient safety work has been conducted since 2005. However, a national MER system is still lacking. The Finnish National Supervisory Authority for Welfare and Health (Valvira) is the national authority that investigates patient safety incidents that have led to severe harm or death because of inappropriate care.^31^ Valvira’s legal mandate is to improve the quality and safety of health care services through the guidance and supervision of registered health care professionals. Valvira undertakes the legitimation and registration of health care professionals and supervises their professional performance after the registration. The postregistration supervision is based mainly on complaints from patients and/or their relatives and notifications from, for example, employer or the police. This study aimed to analyze severe MEs reported to the Valvira in Finland and to evaluate how the documentation of incidents in such a data source applies to learning from errors at the national level.

## METHODS

### Study Material

This study was a retrospective document analysis.^32^ The material consisted of (1) medication-related complaints that Valvira had investigated and closed, and (2) medication-related authoritative statements that Valvira had made for the police of Finland during the period 2013–2017. In the authoritative statements, Valvira assesses the appropriateness of the provided care in cases under inspection by the police to assist in determining whether criminal proceedings should take place.

**FIGURE 1:**
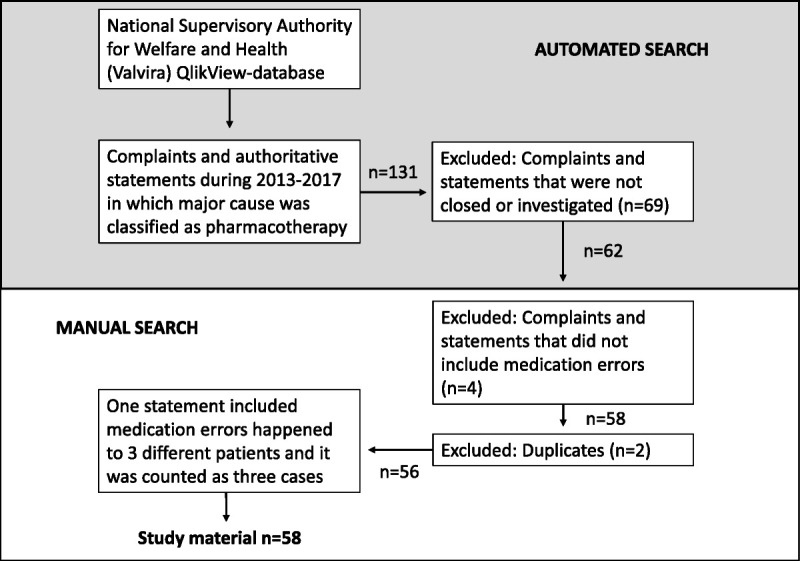
Data inclusion and collection protocol for MEs investigated by the National Supervisory Authority for Welfare and Health (Valvira) in Finland in 2013 to 2017.

The medication-related complaints and statements fulfilling the following inclusion criteria were included in the study: the primary cause was classified as “pharmacotherapy” by Valvira, the case was closed, and Valvira assessed the case to include inappropriate patient care (Fig. 1). The data search was done first in Valvira’s electronic database using the automated search tool and then finalized manually by a researcher (A.T.; Fig. 1). The cases were not included or excluded based on the severity of the outcome to the patient; instead, both cases with actual harm or near miss (the error was noticed and corrected before it reached the patient) were included in the study material. The documentation of the complaints and statements was included in most of the cases: (1) a copy of the patient records and other documents needed for incident evaluation, (2) responses from the professionals involved and/or managers of the health care organization, (3) an external expert (physicians or other specialists) opinion, and (4) the incident report written by the Valvira’s senior medical or legal officer. This incident documentation was qualitative narrative data in nature, and it described the incident and its circumstances, as well as the conclusion of the case. The total material per case varied between 20 and 150 pages.

### Theoretical Framework

The theoretical framework for the study was the system approach to human error and error management to understand the processes leading to severe MEs and harm.^33,34^ This study applied the comprehensive ME definition by the National Coordinating Council for Medication Error Reporting and Prevention, which is also the widely used definition in previous studies.^35,36^

### Data Collection

For collecting the data, a structured data collection form was developed by the research group based on their previous study.^37^ Data from the complaints and statements were collected by a researcher (A.T.) using the data collection form. The data collection form was anonymous and therefore did not include any information on the patients, professionals, or organizations involved in the error cases. The data collection form recorded the following information: (1) patient background information (age, sex), (2) medicines involved in the error, (3) step(s) of the medication process where the error happened, (4) setting (e.g., hospital) where the error happened, (5) professional group(s) involved, (6) harm to the patient, (7) researcher’s assessment of preventability of the error.

### Data Analysis

Harm for the patient in the cases was assessed with 4 categories: death, severe harm, nonsevere harm, and no harm. Harm for the patient was defined as severe when the error had been life-threatening, led to hospitalization or prolonged hospitalization, or caused permanent or significant injury with incapacity.^38^ Medicines were regarded as high-alert medicines if they were present in the lists of the Institute for Safe Medication Practices (ISMP).^17–19^ The preventability of the errors in this study was defined according to the systems approach to human error^33^ and by modifying the definitions used in previous studies^37,39^ (Table 1). Those MEs that had the potential to cause harm for the patients but were noticed before reaching the patient were categorized as prevented.

**TABLE 1:** Definition of the ME Preventability Used in the Study (Modified According to Hallas et al^39^ and Linden-Lahti et al^37^

ME Preventability	Description
Prevented	The error was prevented before it reached the patient.
Likely preventable	There was an existing procedure, operating model, or a guideline, and the error would have been prevented when acting according to it, but it was not followed.
Potentially preventable	There was no existing procedure, operating model, or guideline, but the error could have potentially been prevented from reoccurring with some medication safety development actions.
Unlikely preventable	Error or adverse drug event that was unlikely to be anticipated and would be difficult to prevent to reoccur even with new systemic defenses or other prospective medication risk management actions.

Although the qualitative data documented in the incident reports were carefully read by case, the information of interest was recorded in a structured data collection forms. In cases with difficulties categorizing the data, discrepancies were solved as the consensus of 2 researchers (A.T. and C.L.-L.). The quantified, structured, and categorized data were analyzed using descriptive statistics (frequencies and percentages; Microsoft Excel; Microsoft Corporation, Redmond, Washington).^40^

Cases that included information on the organizations’ actions to prevent reoccurrence of such MEs and improve medication safety were further analyzed. Those medication safety actions were identified, analyzed, and categorized using the Institute for Healthcare Improvement (IHI) Action Hierarchy Template.^41^ According to the action hierarchy, the stronger the preventive action is, the less it is based on human performance.^41,42^

### Research Ethics

The study was conducted with the research permit and supervision of Valvira. Guidelines for research ethics and data protection were followed throughout the research process.^43^

## RESULTS

### Characteristics of MEs

During the period 2013–2017, Valvira received a total of 1654 complaints and statement requests.^44^ In this 5-year study period, 58 cases with MEs fulfilling the study criteria were found from Valvira’s database (Table 2). In the medication process, errors were fatal in 21 cases (36%) and caused severe harm in 9 cases (16%). Nonsevere harm was the end-result of an error in 19 cases (33%). In 3 cases (5%), the error was detected before it reached the patient, and in 6 cases (10%), the researcher was not able to assess the harm level because of the lack of information in the case reports.

**TABLE 2:** Characteristics of MEs (n = 58) Investigated by Valvira During the Period 2013–2017

Characteristic	n	%
Patient sex	58	
Female	34	59
Male	24	41
Patient age, y	58	
0–19	0	0
20–39	4	7
40–59	6	10
60–79	15	26
80–99	33	57
Severity of harm	58	
Death	21	36
Severe harm	9	16
Nonsevere harm	19	33
No harm	3	5
Not able to assess	6	10
Preventability	58	
Likely preventable	39	67
Potentially preventable	14	24
Unlikely preventable	2	4
Prevented	3	5
Error setting*	64	
Assisted living facility	16	25
University hospital	10	16
Primary care ward outside hospital	10	16
Central hospital	10	16
Primary care hospitals	9	14
Public health center	4	6
Home care	3	5
Pharmacy	1	2
Private medical center	1	2
Health care professional involved*	74	
Physician	37	50
Practical nurse	17	23
Nurse	13	18
Student	5	7
Pharmacist	2	3
Medication process phase*	81	
Prescribing	38	47
Administration	15	19
Monitoring	14	17
Dispensing	6	7
Documentation	5	6
Use of medicine by the patient	1	1
Distribution from pharmacy	1	1
Ordering medication from the pharmacy	1	1

*One error process can include several settings, professionals involved, or failures.

Of the patients who had suffered from ME, 59% (n = 34) were female and 41% (n = 24) were male (Table 2). The average age of the patients was 74 years, with a range of 25 to 99 years. A majority (83%; n = 48) of the patients were older than 60 years. According to these data, the ME victim was most likely a woman older than 80 years (n = 25; 43%). In total, 91% (n = 53) of the errors were assessed as likely or potentially preventable, with only 2 cases (3%) resulting in patient death assessed to be unlikely preventable.

A typical setting for an ME was a hospital (in secondary or tertiary care n = 20; in primary care, n = 9; total, n = 29 [45%]), but also settings where older people are mostly cared (e.g., primary care wards outside hospital, assisted living facilities, home care), were highly represented (n = 29; 45%). In 6 cases (10%), 2 different organizations were involved in the ME process, whereas most of the cases (n = 52; 90%) were associated with one organization.

Physicians (n = 37; 50%) were the health care professionals most commonly involved in the investigated ME incidents, followed by practical nurses (n = 17; 23%) and nurses (n = 13; 18%) (Table 2). In 28% (n = 16) of the cases, more than 1 health care professional was involved in the ME process. In 7 cases (12%), Valvira had concluded that the ME was caused by process deficiencies in the organization, not by individual health care professionals’ inappropriate performance. Most of the errors occurred in prescribing (n = 38; 47%), administration (n = 15; 19%), and monitoring (n = 14; 17%) phases of the medication process. In 41% (n = 24) of the cases, the same ME was observed in several phases of the medication use process.

### The Medicines Involved in the Errors

The total number of medicines involved in all error cases (n = 58) was 131, representing 77 different effective substances (Table 3). In these cases, specific effective substances were identified for 126 medicines, whereas for 5 medicines, only the therapeutic ATC group (level 2–3 code) was known.^45^ Nearly half of the cases (n = 26; 45%) included more than 1 effective substance. The top 5 therapeutic groups (ATC levels 2–3) most frequently involved in the errors (n = 58) were antithrombotic agents (n = 17; 13%), opioids (n = 10; 8%), antipsychotics (n = 10; 8%), drugs used in diabetes (n = 8; 6%), and drugs for cardiac therapy (n = 8; 6%). Oxycodone and enoxaparin were found to be the most common specific effective substances associated with the MEs. Both medicines were reported in 7 of 58 cases (Table 3). Errors with enoxaparin were associated with prescribing too high doses, insufficient therapeutic monitoring, or treatment duration. Problems with oxycodone were typically exceeding the prescribed dose when using oral suspension, giving medicine to a wrong patient, wrong administration route, or failure in adjusting the dose according to changes in patient condition.

**TABLE 3:** Medicines (n = 131) Involved in All Error Cases (n = 58) According to Level 2–3 ATC Codes^45^

ATC Group	n (%)	Specific Effective Substance Mentioned in >1 Error Case (n of the Cases)
B01 Antithrombotic agents	17 (13.0)	Enoxaparin (7), warfarin (4), acetylsalicylic acid (3)
N02A Opioids	10 (7.6)	Oxycodone (7), fentanyl (2)
N05A Antipsychotics	10 (7.6)	Quetiapine (4), haloperidol (3)
A10 Drugs used in diabetes	8 (6.1)	Metformin (3)
C01 Cardiac therapy	8 (6.1)	Isosorbide mononitrate (2), isosorbide dinitrate (2), digoxin (2)
N05C Hypnotics and sedatives	7 (5.3)	Temazepam (4)
N05B Anxiolytics	6 (4.6)	Diazepam (3), lorazepam (2)
C03 Diuretics	5 (3.8)	Furosemide (5)
C07 β-blocking agents	5 (3.8)	Metoprolol (3), bisoprolol (2)
N03 Antiepileptics	5 (3.8)	—
A12AX Calcium, combinations	4 (3.1)	Calcium and vitamin D combination (4)
J01 Antibacterials for systemic use	4 (3.1)	—
R03 Drugs for obstructive airway diseases	4 (3.1)	—
H03 Thyroid therapy	3 (2.3)	Levothyroxine (3)
A06 Drugs for constipation	3 (2.3)	—
N01 Anesthetics	3 (2.3)	—
N06D Antidementia drugs	3 (2.3)	—
V03 All other therapeutic subgroups	3 (2.3)	Naloxone (3)
A02 Drugs for acid related disorders	2 (1.5)	—
A07 Antidiarrheals, intestinal anti-inflammatory/anti-infective agents	2 (1.5)	—
H02 Corticosteroids for systemic use	2 (1.5)	—
N02B Other analgesics and antipyretics	2 (1.5)	Paracetamol (2)
N06A Antidepressants	2 (1.5)	—
S01 Ophthalmologicals	2 (1.5)	—
Other	11 (8.4)	—
Total	131	

Only medicines mentioned in >1 error cases are presented according to specific effective substances.

In total, 36% (n = 47) of the effective substances in MEs were identified as high-alert medicines.^17–19^ Effective substances involved in the MEs resulting in severe harm or death of a patient (n = 30) are described in Table 4. Many high-alert medicines are found at the top of the severe ME list (enoxaparin, oxycodone, warfarin), but also other medicines were associated with severe harm or death of the patient.

**TABLE 4:** Effective Substances (n = 78) Involved in MEs That Caused Severe Harm or Death of a Patient (n = 30)

Medicine (n = 78)	n (%)
Enoxaparin*	5 (6.4)
Furosemide	4 (5.1)
Oxycodone*	4 (5.1)
Warfarin*	3 (3.8)
Naloxone	3 (3.8)
Quetiapine	3 (3.8)
Metoprolol	3 (3.8)
Bisoprolol	2 (2.6)
Isosorbide mononitrate	2 (2.6)
Metformin*	2 (2.6)
Digoxin*	2 (2.6)
Diazepam	2 (2.6)
Other	43 (55.1)
Total	78

Only effective substances mentioned in >1 errors are presented with a name.

*Included in the ISMP list of high-alert medicines.^17–19^

The administration route for 130 medicines involved in all errors (n = 58) was identified. The medicines were administered typically orally, intravenously, or subcutaneously (Table 5). Most of the medicines in severe MEs were administered per oral (72%).

**TABLE 5:** Administration Routes of the Medicines Involved in All MEs (n = 58) and MEs That Caused Severe Harm or Death of a Patient (n = 30)

Administration Route of the Medicine	Medicines in All MEs (n = 130), n (%)	Medicines in MEs Causing Severe Harm or Death (n = 81), n (%)
Per oral	89 (69)	58 (72)
Intravenous	15 (12)	7 (9)
Subcutan	13 (10)	10 (12)
Epidural	4 (3)	4 (5)
Inhalation	4 (3)	—
Intramuscular	2 (2)	2 (3)
Ocular	2 (2)	—
Transdermal	1 (1)	—

### Actions Taken in the Organizations After the ME Had Occurred

**FIGURE 2:**
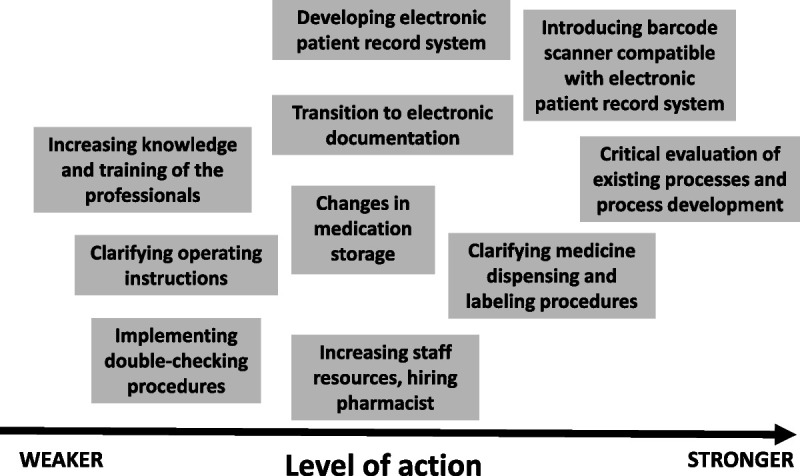
Reported actions taken by health care organizations to improve medication safety after the ME had occurred. The strength of the actions classified according to the IHI Action Hierarchy.^41^

In 60% (n = 35) of the cases (n = 58), the documents available in Valvira included a description of the organization’s changes to their medication processes to prevent the reoccurrence of the errors. Reported organizational changes and/or actions to improve medication safety were multiple, ranging from staff training to introducing technology-based systemic defenses to the processes. A summary of different actions and their level of strength based on the IHI Action Hierarchy^41^ is presented in Figure 2.

## DISCUSSION

### Health Care Authority Data as a Source of Medication Safety Information

This register-based retrospective document analysis was intended to explore MEs reported to a national health care authority Valvira in Finland. Based on our study, the health care authority data proved to be a rich and multidimensional source of medication safety information. A primary reason for its uniqueness is that it provides information on the severe errors that are rare^46^ and may not be reported to other MER systems.^23,25^ Because the authority documentation is descriptive and qualitative, it provides a detailed picture of “what went wrong” and in which phases of the medication process, causing a severe incident. Furthermore, the incident reporting to Valvira is patient-driven; a central patient expectation of a complaint process is that a similar error would not happen to anyone else.^29^ Therefore, our findings complemented with the previous studies recommend using the authority documentation as a source of medication safety information. In countries with well-established MER systems providing structured information, authority documentation could perform as a supplementary data source on severe MEs. The national supervisory authorities’ central role as a provider of medication safety information should also be recognized and established in national and international patient safety improvement policies.

### What Can We Learn From Severe MEs?

Our study revealed that elderly people, particularly those older than 80 years were the most vulnerable to severe MEs investigated by Valvira. In the Finnish population, the proportion of the age group of 60 years or older is approximately 29%.^47^ Although medication treatment in this population is more common, they presented 83% of the patients involved in the severe MEs of this study. Previous studies have reported similar findings, indicating that the effects of MEs are likely to be more harmful to the elderly population with reduced physiological and cognitive functions.^6,7,14,48^ Because the elderly people were prevalent in the Valvira’s data, the medicines involved in the errors represent medicines typically used in the care of this particular age group. However, many of those medicines are also categorized as potentially inappropriate medication used in elderly people.^49^

Many of the top 10 medicines associated with severe errors in this study, such as antithrombotic agents and opioids, have been reported as high-alert medications by the previous studies and the ISMP.^3,6,13,17–19,50^ This finding strengthens the need to adopt effective, evidence-based error safeguarding interventions to different stages of these medicines’ medication process. However, severe errors also occurred with medicines that are not regarded as high-alert medications. This finding may indicate that the severity of the error may be cause of not only the medication itself but also the health status, multimorbidities, and age of the patient, and other systemic contributing factors.^6,15^ Although many studies have emphasized, for example, intravenous administration as a high-risk administration route,^51^ our study also highlights the risk associated with the oral treatment.

The present study demonstrates that severe MEs are a challenge to safe care in all health care settings where medicines are used. To our knowledge, there are only few studies on severe MEs that have included all patient care settings. Our study revealed that assisted living facilities but also primary care wards outside the hospitals and home care were equally prone to severe MEs than hospitals. Those settings are often environments where the elderly population is treated and may lack established safe and high-quality medication processes and even health care staff competency.^52^ This study indicates that preventive medication safety risk management actions in the care of elderly people, and possibly other high-risk populations such as children, should be a high priority for health care settings. In Finland, patient safety challenges in assisted living facilities have been a national crisis reflecting deficiencies in several key areas of safe medication care, such as lack of staff competencies and recourses allocated to elderly care, and often occurring absence of a physician in charge of the entire medication treatment of an individual patient.

This study is in line with the previous studies demonstrating that most of the MEs take place in prescribing and administration stages of the medication process.^7,8,10,13,48^ According to our study supported by similar findings by Panesar et al,^10^ monitoring medication use represents a phase of the medication process that defensive actions should strengthen. The severe incidents in this study also typically included more than 1 ME, many organizations, and several medicines. Like the Human Error model by Reason^33,34^ suggests, severe MEs are often complex processes including many errors and professionals failing to block the error before it causes harm for the patient. There is a need for more qualitative studies to understand the whole process chain behind the severe errors, instead of simple calculations and classifications of the error types to learn effectively from MEs.

### Preventing Severe MEs

As suggested by previous studies, most of the severe MEs in this study were assessed as likely or potentially preventable, providing the health care organizations an opportunity to reduce the reoccurrence of these errors by systems-based defences.^13,53^ More than half of the errors had already led to developing such systems, processes, resources, and competencies. Indeed, it is an encouraging finding that the current severe ME prevention measures seemed to focus on a systems perspective, understanding human errors, and applying medication safety interventions with varying levels of strength, such as adding pharmacist resources or technical solutions.^41,42^ When developing medication safety, it is important to develop medication processes with varying actions and always, when possible, to select the strongest possible defenses.

### Limitations

In this retrospective document analysis, the researchers could not contact the professionals or organizations involved in the errors. Therefore, the possibility existed to misinterpret the free-text data, and all the information to determine comprehensively why the errors happened was not available. The preventability of the errors was especially challenging to evaluate, and the information on the conducted safety development actions in the organizations was, in many cases, missing or incomplete. Also, determining whether one specific ME caused the harm or death of a fragile patient with comorbidity was not always a clear cause-effect relationship. These findings represent key development targets for countries willing to develop the quality and use of their similar data sources for learning from severe MEs. Further research is also needed on the organizational development actions after severe MEs to widen the learning opportunities nationally and internationally.

## CONCLUSIONS

Medication errors reported to a national health care supervisory authority are a valuable and unique information source of severe errors, and these data should be regarded as a part of national incident reporting and learning systems in different countries. High age remains a key risk factor to severe MEs, which may be associated with a wide range of medications including those not typically perceived as high-alert medications or high-risk administration routes. Ensuring comprehensive medication safety of elderly and fragile patients should be a primary focus of all care settings, with an emphasis in primary care and long-term care facilities. Despite being complex processes, severe MEs have a great potential to lead to developing systems, processes, resources, and competencies of health care organizations. In conclusion, learning from severe MEs and finding the most effective process defenses to combat their occurrence remain the challenge of health care systems, nationally and globally.
